# Phosphorylation of Chromosome Core Components May Serve as Axis Marks for the Status of Chromosomal Events during Mammalian Meiosis

**DOI:** 10.1371/journal.pgen.1002485

**Published:** 2012-02-09

**Authors:** Tomoyuki Fukuda, Florencia Pratto, John C. Schimenti, James M. A. Turner, R. Daniel Camerini-Otero, Christer Höög

**Affiliations:** 1 Department of Cell and Molecular Biology, Karolinska Institutet, Stockholm, Sweden; 2 Genetics and Biochemistry Branch, National Institute of Diabetes and Digestive and Kidney Diseases, National Institutes of Health, Bethesda, Maryland, United States of America; 3 Department of Biomedical Sciences, College of Veterinary Medicine, Cornell University, Ithaca, New York, United States of America; 4 Division of Stem Cell Biology and Developmental Genetics, Medical Research Council, National Institute for Medical Research, London, United Kingdom; The Jackson Laboratory, United States of America

## Abstract

Meiotic recombination and chromosome synapsis between homologous chromosomes are essential for proper chromosome segregation at the first meiotic division. While recombination and synapsis, as well as checkpoints that monitor these two events, take place in the context of a prophase I-specific axial chromosome structure, it remains unclear how chromosome axis components contribute to these processes. We show here that many protein components of the meiotic chromosome axis, including SYCP2, SYCP3, HORMAD1, HORMAD2, SMC3, STAG3, and REC8, become post-translationally modified by phosphorylation during the prophase I stage. We found that HORMAD1 and SMC3 are phosphorylated at a consensus site for the ATM/ATR checkpoint kinase and that the phosphorylated forms of HORMAD1 and SMC3 localize preferentially to unsynapsed chromosomal regions where synapsis has not yet occurred, but not to synapsed or desynapsed regions. We investigated the genetic requirements for the phosphorylation events and revealed that the phosphorylation levels of HORMAD1, HORMAD2, and SMC3 are dramatically reduced in the absence of initiation of meiotic recombination, whereas BRCA1 and SYCP3 are required for normal levels of phosphorylation of HORMAD1 and HORMAD2, but not of SMC3. Interestingly, reduced HORMAD1 and HORMAD2 phosphorylation is associated with impaired targeting of the MSUC (meiotic silencing of unsynapsed chromatin) machinery to unsynapsed chromosomes, suggesting that these post-translational events contribute to the regulation of the synapsis surveillance system. We propose that modifications of chromosome axis components serve as signals that facilitate chromosomal events including recombination, checkpoint control, transcription, and synapsis regulation.

## Author Summary

Meiosis is a specialized cell division to generate haploid sperm and eggs. For accurate segregation of homologous chromosomes during the first meiotic division, chromosome synapsis and recombination should be properly established between them during the prophase I stage. Chromosome synapsis and recombination proceed in the context of the meiotic chromosome axis. While studies using knockout mouse models have revealed that chromosome axis components play roles in multiple chromosomal events during mammalian meiosis, it remains to be elucidated how they contribute to the processes. Here, we show that many mammalian meiotic chromosome axis proteins are phosphorylated in a spatially and temporally distinct manner during the prophase I stage. Especially, phosphorylation of HORMAD1 and SMC3 was observed preferentially in chromosomal regions where synapsis has not occurred. Moreover, phosphorylation of HORMAD1 and HORMAD2 was reduced in mutant testicular cells that were defective in recombination initiation or chromosome axis organization. Additionally, the mutant spermatocytes failed to correctly distribute checkpoint proteins that coordinate chromosome synapsis with gene expression and meiotic progression. Thus, it is suggested that phosphorylation of chromosome axis proteins serves as integrative axis marks for the status of events that take place on meiotic chromosomes.

## Introduction

Meiosis is a special type of cell division that gives rise to haploid gametes required for sexual reproduction. To halve the chromosome number, two successive chromosome segregation events follow a single round of DNA replication. At the first stage of meiosis, the leptotene stage of prophase I, recombination is initiated between homologous chromosomes (homologs) by programmed DNA double-strand breaks (DSBs) formed by the SPO11 protein [1]. Recombination is, in some organisms including mice, required for synapsis of homologs [2], [3]. At the zygotene stage of prophase I, homologs come into close proximity and the synaptonemal complex (SC) assembles between the aligned homologs [4], [5]. At the pachytene stage of prophase I, the homologs become fully synapsed by the SCs and repair of a subset of DSBs results in crossover recombination. At the diplotene stage of prophase I, the SCs are disassembled and the homologs undergo desynapsis, now attached to each other only at crossover sites. The physical connections between the homologs, called chiasmata, are essential for correct segregation of the homologs at the anaphase stage of meiosis I [6]. Thus, processes that transform the nature of meiotic chromosomes, such as recombination and synapsis, are executed in a coordinated manner during prophase I.

The integrity of the recombination process and chromosome synapsis during prophase I is monitored by cellular surveillance systems [7]. Checkpoint kinases such as ATM (ataxia telangiectasia mutated) and ATR (ATM and Rad3-related) play key roles in the meiotic surveillance systems in many organisms, including mice. In budding yeast, Mec1 and Tel1, the yeast orthologs of ATR and ATM, respectively, are activated by Spo11-generated DSBs to regulate the pachytene checkpoint that monitors recombination and synapsis [7]. In mammals, deficiencies in recombination or synapsis give rise to meiotic arrest or cell death at the late zygotene or pachytene stage of prophase I [8]–[11]. This checkpoint-like phenomenon is thought to be controlled by MSUC (meiotic silencing of unsynapsed chromatin), a surveillance system that monitors synapsis and causes gene silencing [10]. In MSUC, ATR is recruited to unsynapsed chromosomal regions together with ATR activators, such as BRCA1 and TOPBP1, and induces phosphorylation of histone H2AX (γH2AX) in those regions [10]. This post-translational signal triggers chromatin alterations, leading to transcriptional silencing [10], [12]. The MSUC machinery is proposed to control meiotic progression, by silencing gene expression on the XY chromosomes in male germ cells and by a yet-to-be-determined mechanism in female germ cells [10], [13]–[16]. In contrast to ATR, ATM is dispensable for meiotic surveillance systems including MSUC in mouse meiosis, while it is required for completion and regulation of meiotic recombination [8], [9], [17].

Meiotic recombination and synapsis take place in the context of a prophase I-specific chromosome structure. Chromosomes consisting of two sister chromatids are organized in linear arrays of chromatin loops whose bases are attached to the chromosome axis [4]. The chromosome axis is associated to a single axial chromosome core composed of cohesin complex proteins and cohesin regulators, which promote sister chromatid cohesion and include meiosis-specific cohesin subunits, such as REC8, RAD21L, SMC1β and STAG3, as well as the canonical cohesin subunits (SMC3, SA1/2, SMC1α and RAD21) and cohesin-associated proteins (WAPL and PDS5B) [18]–[23]. The cohesin core serves as a scaffold for the assembly of the axial element (AE) of the SC, a protein structure that promotes additional chromosome axis organization. SYCP2 and SYCP3 are major components of the mammalian AE and are essential for its formation [24], [25]. Proteins harboring a HORMA (Hop1, Rev7 and Mad2) domain represent a third group of chromosome axis proteins in eukaryotes and include the mammalian HORMA domain-containing proteins, HORMAD1 and HORMAD2. In contrast to cohesin complex proteins and AE proteins, HORMAD1 and HORMAD2 bind preferentially to chromosome axes where homologs are not synapsed, such as axes prior to synapsis (unsynapsed) and axes where the SC has disassembled after completion of synapsis (desynapsed) [26], [27]. We have monitored here the phosphorylation status of individual chromosome axis proteins in mouse spermatocytes during prophase I, to better understand the relationship between axis morphogenesis and axis-associated chromosomal events. We report that chromosome axis proteins, such as cohesin complex proteins, AE proteins and HORMA domain-containing proteins, are phosphorylated in a spatially and temporally distinct manner during mammalian meiosis. We suggest that the observed dynamic changes in the phosphorylation pattern of chromosome axis proteins serve as signals that integrate the recruitment of regulatory proteins with the facilitation of chromosomal events that take place on meiotic chromosomes.

## Results

### Chromosome axis proteins are differentially phosphorylated during the prophase I stage of meiosis

**Figure 1:**
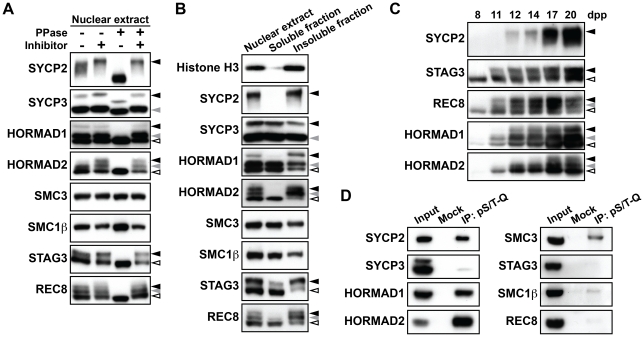
Chromosome axis proteins are phosphorylated during prophase I. (A) Testis nuclear extracts treated with (+) or without (−) phosphatase (PPase) and phosphatase inhibitors (Inhibitor) were probed with antibodies against meiotic chromosome axis components. Phosphatase-sensitive slow-migrating forms are indicated by black and gray arrowheads. (B) Testis nuclear extracts were fractionated into detergent-soluble and detergent-insoluble fractions and analyzed by immunoblotting using antibodies against meiotic chromosome axis components. Histone H3 was used as a control for chromosomal proteins. (C) Testis nuclear extracts from juvenile mice of each age were examined by immunoblotting. (D) Testis nuclear extracts were immunoprecipitated without (Mock) or with the antibody against the phosphorylated S/T-Q motif (pS/T-Q). The immunoprecipitates were electrophoresed on a gradient gel and examined by immunoblotting against chromosome axis proteins. Note that using a gradient gel did not enable separation of phosphorylated and non-phosphorylated forms of chromosome axis proteins.

To examine the phosphorylation status of meiotic chromosome axis components, we performed immunoblotting experiments following SDS-gel separation of mouse testis nuclear extracts. The nuclear extracts were also treated with phosphatase to identify phosphorylated proteins by changes in their gel mobility (Figure 1A and Figure S1). Phosphatase-sensitive protein bands were detected for SYCP2, SYCP3, STAG3, REC8, HORMAD1 and HORMAD2 (Figure 1A, black and gray arrowheads), whereas no obvious mobility shifts were seen for SMC3 and SMC1β (Figure 1A). Thus, most of the chromosome axis proteins that we analyzed are phosphorylated. To examine whether the phosphorylated forms of these proteins are bound to chromosomes, we fractionated testis nuclear extracts. Testis nuclear extracts were treated with detergents containing Triton X-100, and then fractionated by centrifugation into a pellet (insoluble fraction) including chromosome-associated proteins and a supernatant (soluble fraction) containing nucleoplasmic proteins. SYCP2 was found to be highly enriched in the insoluble fraction, whereas the other chromosome axis proteins were found in both fractions (Figure 1B). We found that the phosphorylated forms of HORMAD1, HORMAD2, STAG3 and REC8 were preferentially bound to the chromosome, by comparing the gel mobility of the protein bands in the insoluble fraction to those in the nuclear extracts. In contrast, the phosphorylated forms of SYCP3 appeared at similar levels in both fractions. Thus, chromosome axis proteins bound to chromosomes are frequently phosphorylated.

We next analyzed the timing with which phosphorylation of chromosome axis proteins takes place. We used testis nuclear extracts of juvenile mice, in which a synchronous first wave of spermatogenesis occurs. As shown in Figure 1C, the phosphorylated forms of SYCP2, STAG3, REC8 and HORMAD1 were detected as early as 11 or 12 days postpartum (dpp), corresponding to the leptotene and early zygotene stages of prophase I. For HORMAD2, a phosphorylated form of this protein was first seen at 12 dpp (Figure 1C, gray arrowhead). In addition, a second phosphorylated form appeared at 17 dpp, corresponding to the late pachytene stage (Figure 1C, black arrowhead), suggesting that phosphorylation of HORMAD2 occurs in a temporally-regulated manner.

To gain insights into the nature of the kinases responsible for the observed phosphorylation events targeting chromosome axis proteins, we used an anti-pS/T-Q antibody that recognizes a phosphorylated serine or threonine followed by a glutamine residue, a consensus target sequence for ATM /ATR (S/T-Q motif). Testis nuclear extracts were subjected to immunoprecipitation with the anti-pS/T-Q antibody, and the immunoprecipitates were probed for chromosome axis proteins by immunoblotting. We detected strong protein bands representing SYCP2, HORMAD1 and HORMAD2 in the immunoprecipitates, suggesting that phosphorylation of these proteins occurs at an S/T-Q motif (Figure 1D). We also detected a relatively strong signal for SMC3 in the immunoprecipitates (Figure 1D), implying that this chromosome axis protein is also phosphorylated at an S/T-Q motif despite the absence of a detectable shift in gel mobility (Figure 1A). We saw little or no signal in the anti-pS/T-Q immunoprecipitates for STAG3, SMC1β, REC8 and SYCP3 (Figure 1D), suggesting that these proteins are phosphorylated at other motifs than the S/T-Q motif. Altogether, our results suggest that multiple kinases with different motif-specificity contribute to phosphorylation of chromosome axis proteins.

### The Ser^375^-phosphorylated form of HORMAD1 is restricted to unsynapsed chromosomal axes

**Figure 2:**
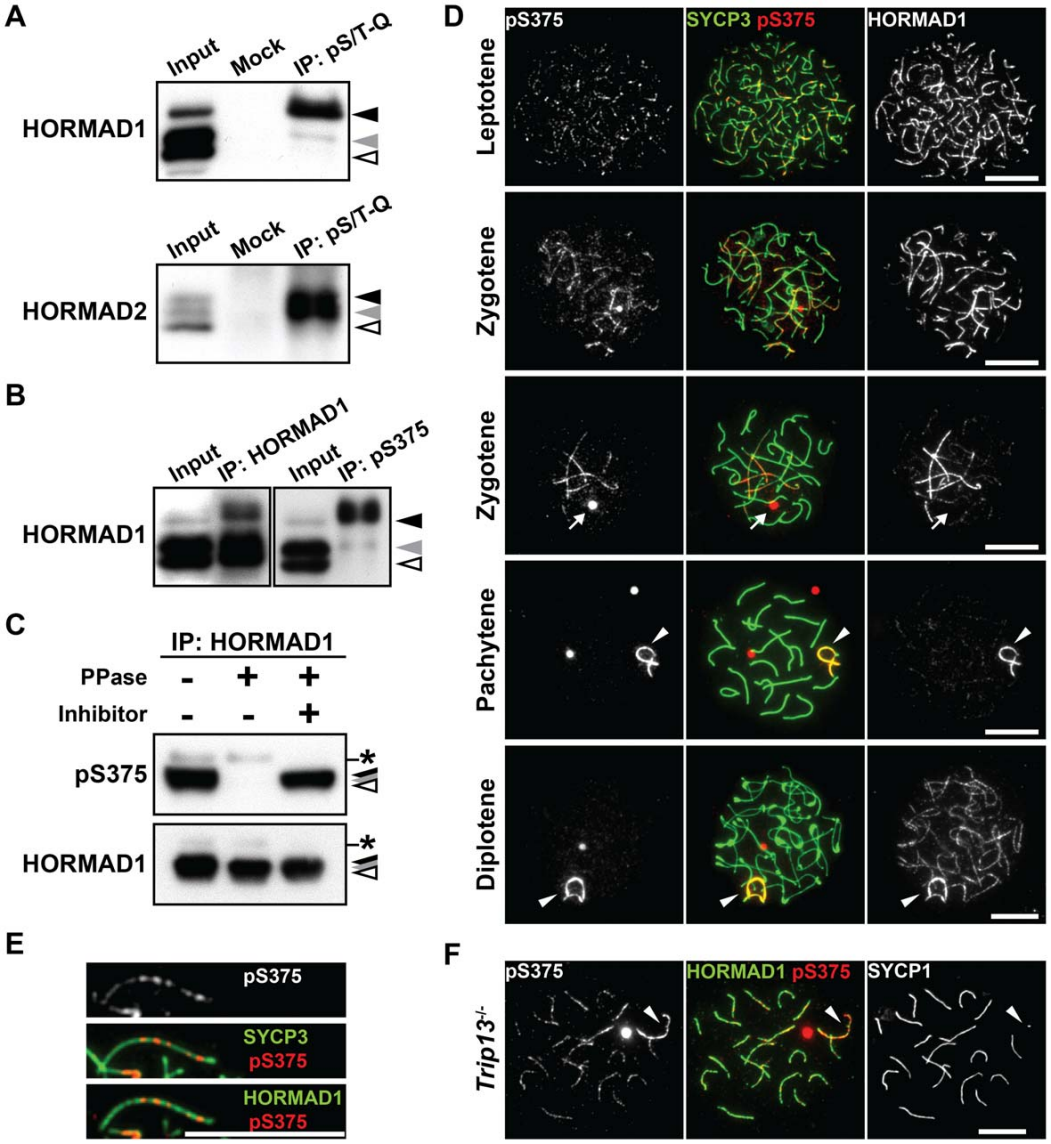
HORMAD1 is phosphorylated at Ser^375^ on unsynapsed chromosomes.

We next investigated the phosphorylation events that target HORMAD1 and HORMAD2 in more detail. Immunoprecipitates of the anti-pS/T-Q antibody were examined using gel conditions that provided better resolution than that seen in Figure 1D, identifying one band strongly labeled by the anti-HORMAD1 antibody (Figure 2A, black arrowhead) and two bands labeled by the anti-HORMAD2 antibody (Figure 2A, black and gray arrowheads). The enrichment of the slowest-migrating phosphorylated form of HORMAD1 (Figure 2A, black arrowhead) suggests that two phosphorylated forms of HORMAD1 exist, one that is phosphorylated primarily at a non-S/T-Q site and one that is phosphorylated at multiple sites containing an S/T-Q site. In contrast, the observation that both phosphorylated forms of HORMAD2 were enriched in the anti-pS/T-Q immunoprecipitates (Figure 2A, black and gray arrowheads) suggests that the both forms of HORMAD2 are phosphorylated at an S/T-Q site(s).

Mouse HORMAD1 and HORMAD2 contain several S/T-Q motifs, including the Ser^375^-Gln^376^ motif in the C-terminal region of HORMAD1 that is highly conserved in vertebrate HORMAD1 proteins (data not shown). Based on this information, we generated a peptide antibody against the Ser^375^-phosphorylated form of HORMAD1 (anti-pS375). Immunoprecipitation and immunoblotting experiments using the anti-pS375 antibody showed that HORMAD1 is phosphorylated at Ser^375^ in testis nuclear extracts (Figure 2B and 2C).

To examine the chromosomal localization of the Ser^375^-phosphorylated form of HORMAD1, nuclear spreads of mouse testicular cells were immunostained using the anti-pS375 antibody. The Ser^375^-phosphorylated form of HORMAD1 was first detectable as series of small foci along the chromosome axes in leptotene spermatocytes, temporally coinciding with loading of HORMAD1 onto the entire chromosome axis, as labeled by the regular anti-HORMAD1 antibody (Figure 2D). The Ser^375^-phosphorylated form of HORMAD1 appeared as discontinuous lines composed of small foci on HORMAD1-labelled unsynapsed chromosome axes during zygotene (Figure 2D and 2E). In pachytene and diplotene spermatocytes, the Ser^375^-phosphorylated form of HORMAD1 overlapped with HORMAD1 at unsynapsed chromosome axes of the XY chromosomes (Figure 2D). Strikingly, whereas the anti-HORMAD1 antibody also labeled desynapsed chromosomal regions that appear at the diplotene stage [26], [27], the anti-pS375 antibody did not (Figure 2D). To confirm this staining pattern, we examined the localization of the Ser^375^-phosphorylated form of HORMAD1 in oocytes during prophase I. We observed that the anti-pS375 antibody labeled series of foci along unsynapsed chromosomal regions in these cells, but notably did not label synapsed or desynapsed regions of chromosomes (Figure S2A).

Depletion of HORMAD1 from the synapsed chromosome axes requires the TRIP13 AAA-ATPase [27]. We therefore examined the chromosomal distribution of the Ser^375^-phosphorylated form of HORMAD1 in a *Trip13* mutant. We observed that the anti-pS375 antibody, in contrast to the situation in wild-type spermatocytes, also labeled discontinuous lines along the chromosome axes of synapsed autosomes in the mutant spermatocytes (89/100 pachytene cells) (Figure 2F). Taken together, our data show that HORMAD1 is phosphorylated at Ser^375^, that the Ser^375^-phosphorylated form of HORMAD1 is restricted to unsynapsed chromosomes in wild-type meiocytes and that TRIP13 facilitates the depletion of the Ser^375^-phosphorylated form of HORMAD1 from synapsed chromosomes.

### The Ser^1083^-phosphorylated form of SMC3 is preferentially associated with unsynapsed chromosomal regions

**Figure 3:**
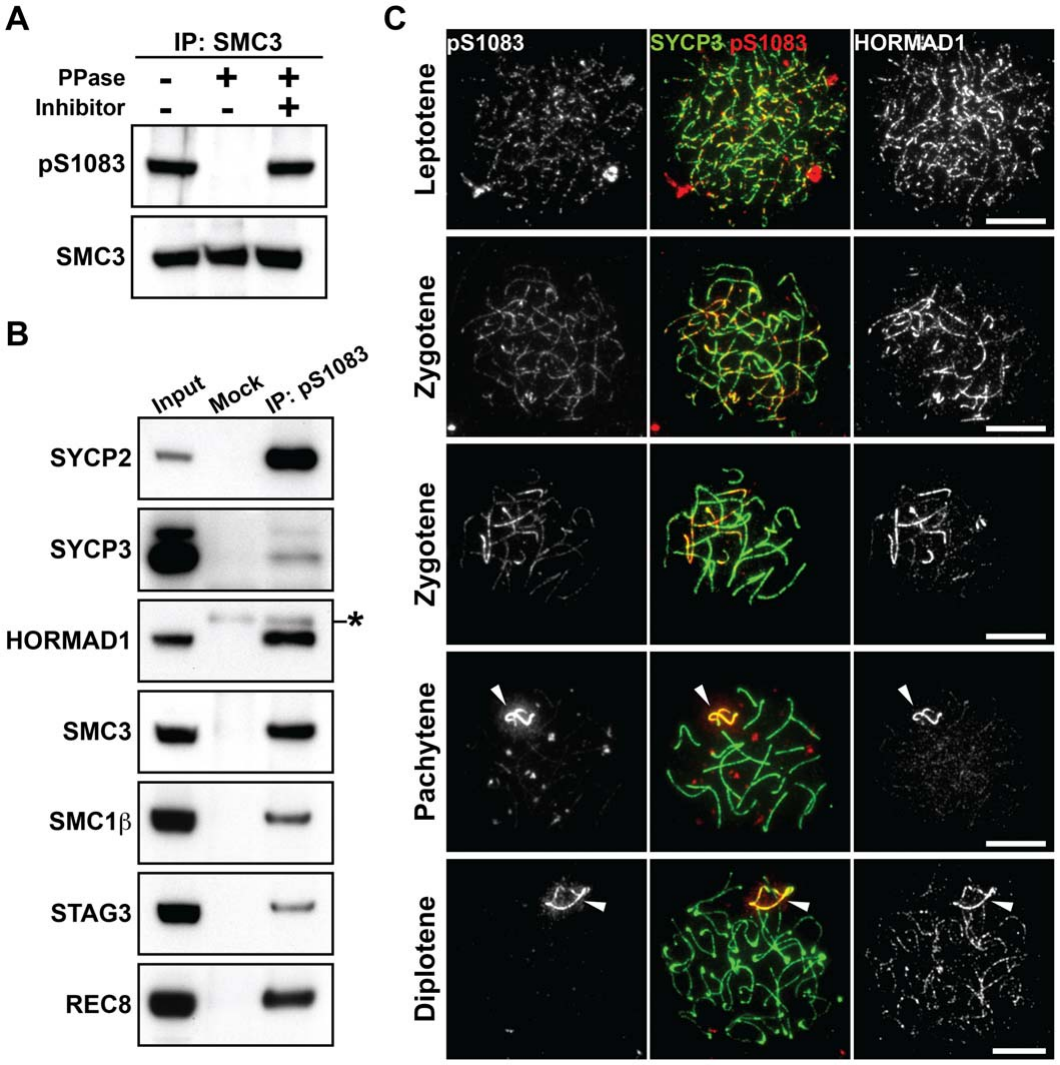
SMC3 is phosphorylated at Ser^1083^ during prophase I. (A) Testis nuclear extracts were immunoprecipitated with the anti-SMC3 antibody, followed by treatment with (+) or without (−) phosphatase (PPase) and phosphatase inhibitors (Inhibitor). 80% of the immunoprecipitated SMC3 and the rest were separated on a gradient gel and immunoblotted with antibodies against the Ser^1083^-phosphorylated form of SMC3 (pS1083) and normal SMC3, respectively. (B) Testis nuclear extracts were immunoprecipitated without (Mock) or with the anti-pS1083 antibody. The immunoprecipitates were probed with antibodies against meiotic chromosome axis components. The asterisk marks a non-specific band. (C) Nuclear spreads of spermatocytes were labeled with anti-pS1083, anti-SYCP3 and anti-HORMAD1 antibodies. Arrowheads indicate the XY bivalent. Bars, 10 µm.

We detected SMC3 in the anti-pS/T-Q immunoprecipitates of testis nuclear extracts (Figure 1D). SMC3 is known to be phosphorylated in mammalian cells at an S/T-Q motif, the Ser^1083^-Gln^1084^ motif, in response to DNA damage [28]. Indeed, immunoprecipitation of SMC3 from testis nuclear extracts followed by immunoblotting using a selective antibody for the Ser^1083^-phosphorylated form of SMC3 (anti-pS1083) identified a protein band in a phosphatase-sensitive manner (Figure 3A). Since SMC3 is expressed in both mitotic and meiotic cells, we addressed whether phosphorylation of SMC3 at Ser^1083^ occurs in the context of the meiotic chromosome axis. Indeed, we found several meiosis-specific cohesin components and AE proteins to be co-immunoprecipitated from testis nuclear extracts with the Ser^1083^-phosphorylated form of SMC3 (Figure 3B). In addition, the anti-pS1083 signal increased when the first wave of spermatogenesis reached the leptotene stage (Figure S3A). Next, nuclear spreads of mouse spermatocytes and oocytes were immunostained using the anti-pS1083 antibody (Figure 3C, Figures S3B and S2B). The Ser^1083^-phosphorylated form of SMC3 was first detectable as foci on chromosome axes in leptotene cells (Figure 3C, Figures S3B and S2B). The Ser^1083^-phosphorylated form of SMC3 was present on both synapsed and unsynapsed chromosomal regions at early zygotene (Figure 3C), whereas the signal intensity increased preferentially at unsynapsed chromosomal regions during late zygotene (Figure 3C, Figures S3B and S2B). In pachytene and diplotene spermatocytes, the Ser^1083^-phosphorylated form of SMC3 accumulated on the XY chromosomes (Figure 3C and Figure S3B). Thus, the Ser^1083^-phosphorylated form of SMC3 is preferentially associated with unsynapsed chromosomes.

### Phosphorylation of HORMAD1 and HORMAD2 partially depends on BRCA1 but not on ATM

**Figure 4:**
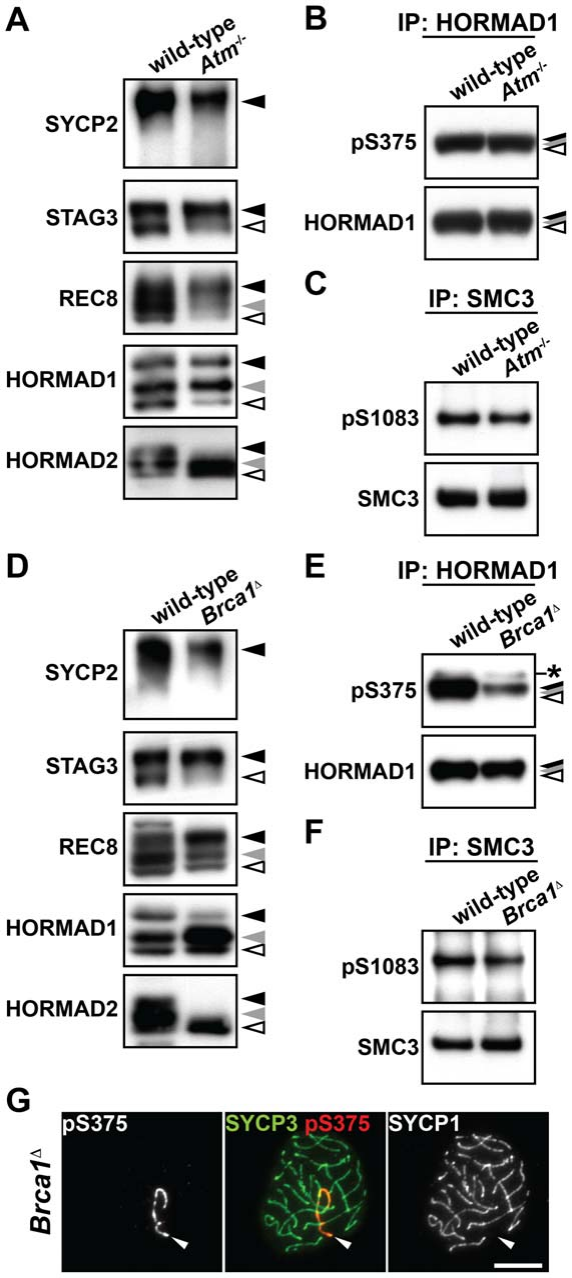
Phosphorylation of chromosome axis proteins in the absence of a checkpoint protein. (A and D) The insoluble fraction of testis nuclear extracts was prepared from *Atm* ^−/−^ (A) and *Brca1* ^Δ11/Δ11^ *Trp53* ^+/−^ (*Brca1^Δ^*) (D) males and probed with antibodies against meiotic chromosome axis components. (B and E) Testis nuclear extracts from *Atm* ^−/−^ (B) and *Brca1* ^Δ11/Δ11^ *Trp53* ^+/−^ (E) males were immunoprecipitated with the anti-HORMAD1 antibody. 80% of the immunoprecipitated HORMAD1 and the rest were separated on a gradient gel and immunoblotted with anti-pS375 and anti-HORMAD1 antibodies, respectively. The asterisk marks a non-specific band. (C and F) Testis nuclear extracts from *Atm* ^−/−^ (C) and *Brca1* ^Δ11/Δ11^ *Trp53* ^+/−^ (F) males were immunoprecipitated with the anti-SMC3 antibody. 80% of the immunoprecipitated SMC3 and the rest were separated on a gradient gel and immunoblotted with anti-pS1083 and anti-SMC3 antibodies, respectively. (G) Nuclear spreads of *Brca1* ^Δ11/Δ11^ *Trp53* ^+/−^ pachytene spermatocytes were labeled with anti-pS375, anti-SYCP3 and anti-SYCP1 antibodies. Arrowheads indicate the XY bivalent. Bar, 10 µm.

We have identified a set of phosphorylation events that target HORMAD1 and SMC3 localized at unsynapsed chromosomal regions and shown that they are phosphorylated at an S/T-Q motif, a known motif for ATM/ATR kinases. We therefore investigated the role of these kinases in phosphorylation of chromosome axis proteins. Nuclear extracts were prepared from the testes of *Atm* ^−/−^ mice and the occurrence of the phosphorylated forms of chromosome axis proteins in the insoluble fraction was analyzed. We found that SYCP2, STAG3, REC8 and HORMAD1 are phosphorylated in the *Atm* ^−/−^ testis nuclear extracts (Figure 4A). We also detected the Ser^375^-phosphorylated form of HORMAD1 and the Ser^1083^-phosphorylated form of SMC3 in the *Atm* ^−/−^ testis extracts (Figure 4B and 4C), as well as in the *Atm* ^−/−^ spermatocytes (Figure S4). We observed a reduced intensity of the slowest-migrating form of HORMAD2 (Figure 4A, black arrowhead). However, since this phosphorylated form of HORMAD2 occurs at the late pachytene stage (Figure 1C), the reduced intensity of this band in the *Atm* ^−/−^ testis extracts is most likely due to the observed loss of germ cells that takes place at the pachytene stage in *Atm* ^−/−^ male mice [8], [29]. Therefore, we conclude that ATM is dispensable for phosphorylation of chromosome axis proteins prior to the pachytene stage.

ATR is localized to unsynapsed chromosomal axes [10]. We found that the distribution of ATR is similar to that of the Ser^375^-phosphorylated form of HORMAD1 from late zygotene to diplotene (Figure S5). To examine if ATR phosphorylates chromosome axis proteins during prophase I, we took advantage of the fact that BRCA1 is required for a subset of ATM/ATR-dependent phosphorylation events [30] and that BRCA1 facilitates the proper distribution of ATR at unsynapsed chromosomal regions during prophase I in meiocytes [13], [31]. We prepared nuclear extracts from testes of *Brca1* ^Δ11/Δ11^ *Trp53* ^+/−^ males, which express a mutated BRCA1 protein that lacks a protein domain encoded by exon 11. The mutated BRCA1 protein fails to correctly distribute recombination proteins to repair sites and ATR to unsynapsed chromosomal regions in spermatocytes [13], [31], [32]. Immunoblotting experiments of the insoluble fraction prepared from the mutant testis nuclear extracts identified the phosphorylated forms of SYCP2, STAG3 and REC8, as well as the Ser^1083^-phosphorylated form of SMC3 (Figure 4D and 4F). In contrast, the intensities of the bands representing the slowest-migrating form of HORMAD1 (Figure 4D, black arrowhead), the Ser^375^-phosphorylated form of HORMAD1 (Figure 4E) and the two slow-migrating forms of HORMAD2 (Figure 4D, black and gray arrowheads) were partially decreased in this mutant. By immunostaining of the mutant pachytene spermatocytes, the Ser^375^-phosphorylated form of HORMAD1 was detected as discontinuous lines on unsynapsed axes of the XY chromosomes (50/50 pachytene cells) (Figure 4G). These findings suggest that the bulk of HORMAD1 phosphorylation is independent of ATR recruited to unsynapsed axes by the MSUC pathway and that BRCA1-regulated ATR may be required for efficient activation or maintenance of phosphorylation of HORMAD1 and HORMAD2 at the unsynapsed chromosome axis.

### SPO11 is required for normal levels of phosphorylation of HORMAD1, HORMAD2, and SMC3

**Figure 5:**
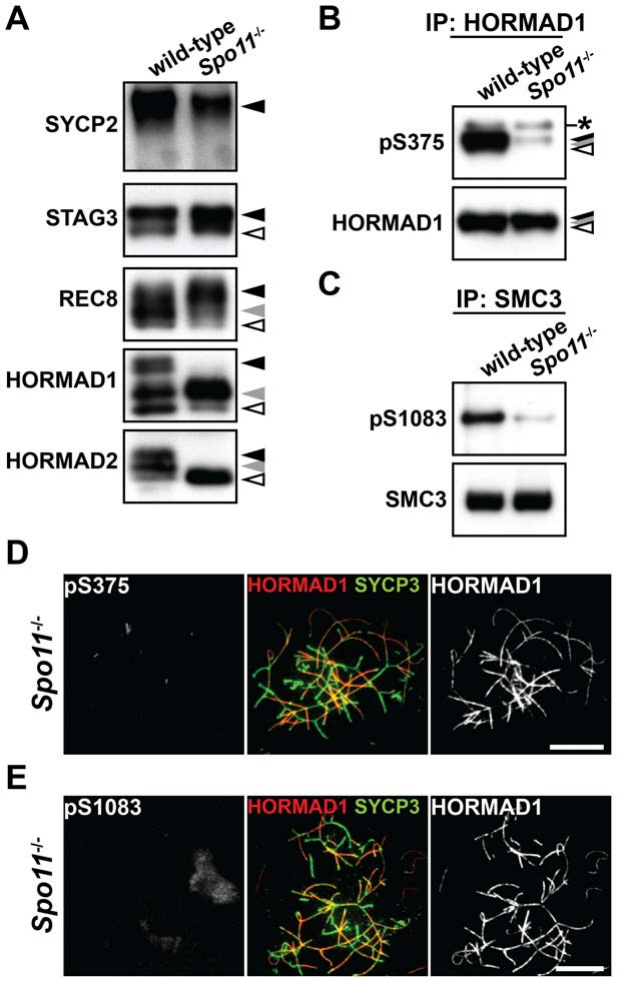
Phosphorylation of HORMAD1, HORMAD2, and SMC3 is highly dependent on SPO11. (A) The insoluble fraction of testis nuclear extracts was probed with antibodies against meiotic chromosome axis components. (B) The Ser^375^-phosphorylated form of HORMAD1 was examined as in Figure 4B. (C) The Ser^1083^-phosphorylated form of SMC3 was examined as in Figure 4C. (D) Nuclear spreads of *Spo11* ^−/−^ zygotene-like spermatocytes were labeled with anti-pS375, anti-SYCP3 and anti-HORMAD1 antibodies. (E) Nuclear spreads of *Spo11* ^−/−^ zygotene-like spermatocytes were labeled with anti-pS1083, anti-SYCP3 and anti-HORMAD1 antibodies. Bars, 10 µm.

To explore the relationship between phosphorylation of chromosome axis proteins and meiotic recombination, we examined the phosphorylation status of chromosome axis proteins in *Spo11* ^−/−^ testicular cells. SPO11-induced DSBs are required for the initiation of meiotic recombination. The phosphorylated forms of SYCP2, STAG3 and REC8 were detected in the insoluble fraction of testis nuclear extracts prepared from *Spo11* ^−/−^ mice, showing that *Spo11* is dispensable for phosphorylation of these proteins (Figure 5A). In contrast, the slowest-migrating form of HORMAD1 (Figure 5A, black arrowhead) and the two slow-migrating forms of HORMAD2 (Figure 5A, black and gray arrowheads) were not observed in the *Spo11* ^−/−^ mutant. Furthermore, a considerably reduced signal was seen for the anti-pS375 antibody for HORMAD1 (Figure 5B) and the anti-pS1083 antibody for SMC3 (Figure 5C) in *Spo11* ^−/−^ mutant testes. We also analyzed the phosphorylation status of HORMAD1 and SMC3 by immunostaining *Spo11* ^−/−^ spermatocytes. Most of the chromosomes in *Spo11* ^−/−^ spermatocytes remain unsynapsed due to lack of recombination, as visualized by intense HORMAD1 labeling on unsynapsed axes in these cells (Figure 5D and 5E). Importantly, the Ser^375^-phosphorylated form of HORMAD1 and the Ser^1083^-phosphorylated form of SMC3 were hardly detected on the axis of unsynapsed chromosomal regions (Figure 5D and 5E). Thus, SPO11 is critically important for phosphorylation of HORMAD1 at Ser^375^, SMC3 at Ser^1083^ and HORMAD2.

### Efficient phosphorylation of HORMAD1 and HORMAD2 requires SYCP3

**Figure 6:**
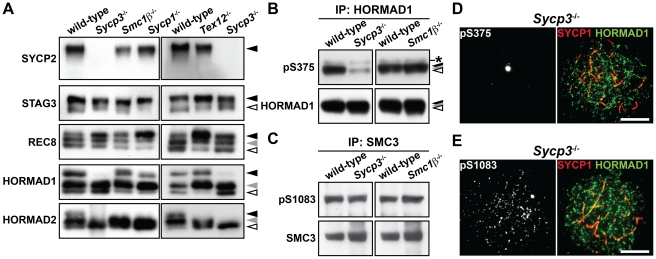
Phosphorylation of HORMAD1 is reduced in the absence of SYCP3. (A) The insoluble fraction of testis nuclear extracts was probed with antibodies against meiotic chromosome axis components. (B) The Ser^375^-phosphorylated form of HORMAD1 was examined as in Figure 4B. (C) The Ser^1083^-phosphorylated form of SMC3 was examined as in Figure 4C. (D) Nuclear spreads of *Sycp3* ^−/−^ zygotene-like spermatocytes were labeled with anti-pS375, anti-SYCP1 and anti-HORMAD1 antibodies. (E) Nuclear spreads of *Sycp3* ^−/−^ zygotene-like spermatocytes were labeled with anti-pS1083, anti-SYCP1 and anti-HORMAD1 antibodies. Bars, 10 µm.

To address the relationship between the phosphorylation of axis proteins and chromosome axis organization, we examined the phosphorylation status of chromosome axis proteins in three SC-deficient mutants, *Sycp1* ^−/−^, *Tex12* ^−/−^ and *Sycp3* ^−/−^, as well as in a mutant deficient for a cohesin complex protein, *Smc1β* ^−/−^. SYCP1 and TEX12 are components of the central region of the SC, a structure that is essential for chromosome synapsis [33], [34], whereas SMC1β is a meiosis-specific cohesin subunit that contributes to chromosome organization and synapsis [35]. As shown in Figure 6A, the phosphorylated forms of SYCP2, STAG3, REC8 and HORMAD1 were detected in the insoluble fraction of *Sycp1* ^−/−^, *Tex12* ^−/−^ and *Smc1β* ^−/−^ testis nuclear extracts. The intensity of the slowest-migrating band of REC8 was increased in the *Sycp1* ^−/−^ and *Tex12* ^−/−^ mutants (Figure 6A, black arrowhead). We also detected the Ser^375^-phosphorylated form of HORMAD1 in *Sycp1* ^−/−^, *Tex12* ^−/−^ and *Smc1β* ^−/−^ spermatocytes (Figure S6). The slowest-migrating form of HORMAD2 that appears at the late pachytene stage was not detected in these three mutants (Figure 6A, black arrowhead), most likely explained by the fact that spermatogenesis arrests at late zygotene or early pachytene in these three mutants [33]–[35]. Taken together, SYCP1, TEX12 and SMC1β are dispensable for phosphorylation of chromosome axis proteins prior to the pachytene stage.

In the *Sycp3* mutant, which does not form AEs and displays synapsis defects [24], [36], the immunoblotting signal of SYCP2 could not be detected in the insoluble fraction of testis nuclear extracts (Figure 6A), consistent with the fact that SYCP2 is not loaded onto the chromosome axis in this mutant [37]. Importantly, the slowest-migrating form of HORMAD1 (Figure 6A, black arrowhead) and the two slow-migrating forms of HORMAD2 (Figure 6A, black and gray arrowheads) were reduced in the absence of SYCP3. We confirmed the reduced level of HORMAD1 phosphorylation in the *Sycp3* mutant by immunoblotting (Figure 6B) and immunostaining (Figure 6D and Figure S7A) using the anti-pS375 antibody. In contrast, the Ser^1083^-phosphorylated form of SMC3 was detectable in the *Sycp3* mutant in both assays (Figure 6C, 6E and Figure S7C). These results show that SYCP3 is required for efficient phosphorylation of HORMAD1 at Ser^375^ and HORMAD2.

### *Sycp3* ^−/−^ spermatocytes are defective in distributing ATR to unsynapsed chromosomal regions

**Figure 7:**
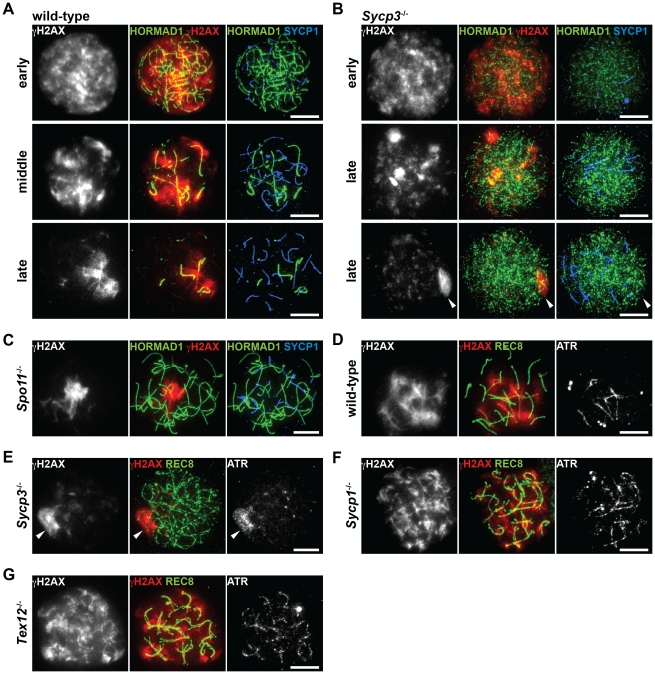
Distribution of ATR at unsynapsed chromosomal regions is impaired in the absence of SYCP3. (A–C) Nuclear spreads of wild-type (A), *Sycp3* ^−/−^ (B) and *Spo11* ^−/−^ (C) zygotene-like spermatocytes were labeled with anti-γH2AX, anti-HORMAD1 and anti-SYCP1 antibodies. (D–G) Nuclear spreads of wild-type (D), *Sycp3* ^−/−^ (E), *Sycp1* ^−/−^ (F) and *Tex12* ^−/−^ (G) zygotene-like spermatocytes were labeled with anti-γH2AX, anti-REC8 and anti-ATR antibodies. Arrowheads indicate the position of the pseudo-sex body-like staining of γH2AX. Bars, 10 µm.

It was recently reported that HORMAD1 is required for loading the MSUC machinery, including ATR and γH2AX, onto the chromosome [16], [38]. To find out if phosphorylation of HORMAD1 and HORMAD2 has a role in chromosome targeting of the MSUC machinery, we analyzed the distribution of γH2AX and ATR in *Sycp3* ^−/−^ spermatocytes, in which phosphorylation of HORMAD1 and HORMAD2 is impaired (Figure 6). In wild-type spermatocytes, SPO11-formed DSBs at the leptotene stage trigger a first wave of γH2AX mediated by ATM, a γH2AX signal that starts to fade away at the early zygotene stage [8], [17]. Subsequently, a second wave of γH2AX emerges during the zygotene stage, phosphorylation of H2AX now mediated by ATR as part of the MSUC pathway that targets unsynapsed chromosomes [17]. Thus, unsynapsed chromosomal regions in wild-type spermatocytes, whose axes are marked by HORMAD1, are labeled with γH2AX during the zygotene stage (Figure 7A and 7D) and also the unsynapsed AEs of the XY chromosomes at the pachytene and diplotene stages (data not shown) [39]. *Sycp3* ^−/−^ spermatocytes are eliminated at a late zygotene stage or an early pachytene stage, and as consequence of this, many chromosomes in the mutant cells remain partially unsynapsed [24], [40]. In *Sycp3* ^−/−^ spermatocytes, the first wave of γH2AX at the leptotene stage took place as seen in wild-type spermatocytes, and the γH2AX signal began to disappear at the early zygotene stage (Figure 7B, top panels). Importantly, in *Sycp3* ^−/−^ cells at a late stage of zygotene, γH2AX failed to become localized to the unsynapsed chromosomes as seen in wild-type zygotene cells. Instead, the γH2AX signal in the mutant cells was localized to restricted domains (Figure 7B, middle panels) or displayed a pseudo-sex-body-like staining pattern (Figure 7B, bottom panels). The pseudo-sex body is a chromosomal domain seen in *Spo11* ^−/−^ zygotene-like spermatocytes (Figure 7C), within which the MSUC machinery accumulates [14], [17], [39]. As seen in *Spo11* ^−/−^ spermatocytes [14], ATR accumulated in the pseudo-sex-body-like domain in *Sycp3* ^−/−^ spermatocytes (Figure 7E). These results show that ATR and γH2AX fail to correctly accumulate at unsynapsed chromosomal regions in *Sycp3* ^−/−^ spermatocytes, as well as in *Spo11* ^−/−^ spermatocytes.

To exclude the possibility that mislocalization of γH2AX and ATR in the *Sycp3* ^−/−^ and *Spo11* ^−/−^ mutants is due to a synapsis defect in these mutants, we also examined the distribution of these markers in *Sycp1* ^−/−^ and *Tex12* ^−/−^ spermatocytes. In these mutant spermatocytes, where meiosis does not proceed beyond the pachytene stage, γH2AX and ATR were observed on the entire length of unsynapsed chromosomes (Figure 7F and 7G) and did not display a pseudo-sex-body-like staining pattern. These results show that the distribution of the MSUC machinery and phosphorylation of HORMAD1 and HORMAD2 are normal despite absence of synapsis. Our results reveal that SYCP3 and SPO11 contribute both to phosphorylation of HORMAD1 and HORMAD2 and to the process through which ATR becomes correctly distributed to unsynapsed chromosomes. Possibly, it is the phosphorylated forms of HORMAD1 and HORMAD2 that mediate the distribution of the MSUC machinery among unsynapsed chromosomes.

## Discussion

### Meiotic chromosome axis proteins are phosphorylated

We show here that a large number of chromosome axis proteins are phosphorylated during the prophase I stage of mouse meiosis. This includes HORMA domain-containing proteins (HORMAD1 and HORMAD2) and components of the cohesin complex (SMC3, STAG3 and REC8) and the AE (SYCP2 and SYCP3), similar to what has been shown previously for some individual mammalian chromosome axis proteins [26], . Chromosome axis proteins are intimately involved in several critical meiotic processes including sister chromatid cohesion, chromosome organization, recombination, synapsis and checkpoint control [16], [24], [35], [38], [45]–[47]. What is the role of the post-translational modifications added to the chromosome axis proteins? They could promote dissociation of proteins from the chromosome axis, in analogy with the displacement of the cohesin complex that occurs in response to phosphorylation at the prophase stage of mitosis [48]. We consider this explanation unlikely however, as phosphorylation of chromosome axis proteins during meiosis starts at an early stage of prophase I, not coinciding with their displacement from the chromosome axis. Phosphorylation of chromosome axis proteins could act more directly to promote different meiotic processes. Supporting this, phosphorylation of the yeast HORMA-domain containing protein, Hop1 in *S. cerevisiae*, is required for the prevention of inter-sister recombination and the pachytene checkpoint [49], while elimination of phosphorylation sites within Rec8 in *S. cerevisiae* causes defects in recombination and synapsis during prophase I [50]. To gain more insight into the functional consequences of the phosphorylation of various chromosome axis proteins during meiosis, we have focused on the role of the phosphorylation events that target SMC3, HORMAD1 and HORMAD2.

### Phosphorylation of SMC3 occurs at unsynapsed chromosomal regions and depends on recombination

In mouse spermatocytes, SMC3 localizes to the meiotic chromosome axis irrespective of the status of chromosome synapsis (Figure S3B) [51]. We found that the Ser^1083^-phosphorylated form of SMC3 is preferentially associated with unsynapsed chromosomal regions but not with synapsed or desynapsed regions from late zygotene to diplotene, similar to the Ser^375^-phosphorylated form of HORMAD1. Phosphorylation of SMC3 at Ser^1083^ depends on SPO11 but is not affected in the absence of full-length BRCA1 and SYCP3, indicating that SMC3 is regulated differently from HORMAD1 and HORMAD2. Moreover, the Ser^1083^-phosphorylated form of SMC3 was detected on both synapsed and desynapsed chromosomes during early zygotene, in contrast to the Ser^375^-phosphorylated form of HORMAD1, which is not detected in synapsed regions. Probably, TRIP13-mediated displacement of HORMAD1 from synapsed chromosome axes enables more strictly regulated localization of HORMAD1 phosphorylation in unsynapsed chromosomal regions.

**Figure 8:**
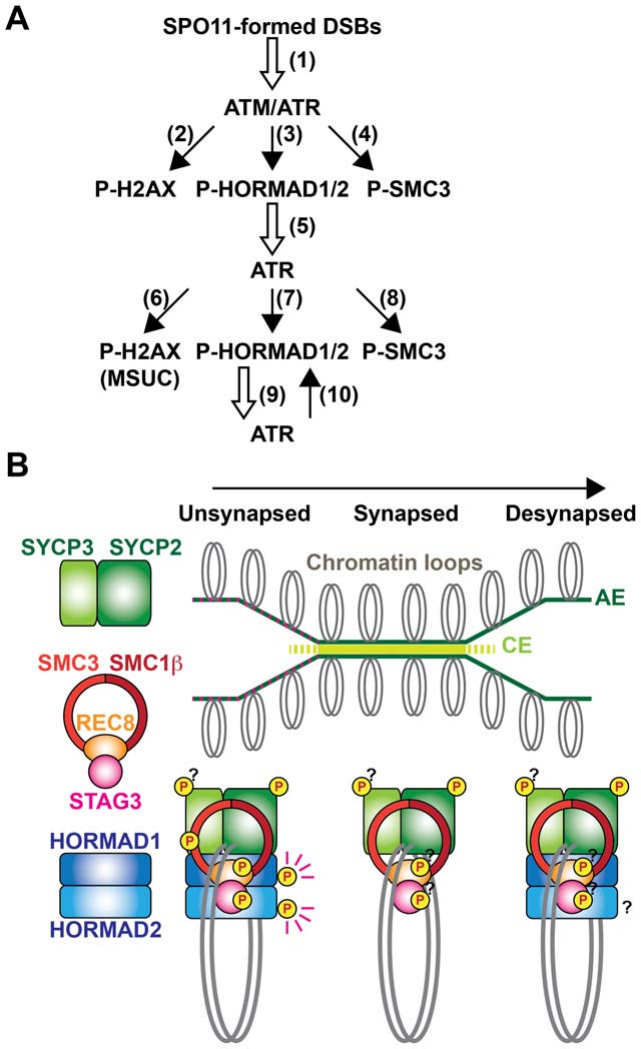
Chromosomal regions are marked by compositional differences and modification status of axis proteins. (A) Schematic representation of the model for regulation of phosphorylation of meiotic chromosomal proteins at S/T-Q motifs. In response to SPO11-formed DSBs (arrow 1), ATM phosphorylates histone H2AX (arrow 2) and ATR phosphorylates HORMAD1/2 (arrow 3) and SMC3 (arrow 4). Phosphorylated HORMAD1/2 serves as a marker for unsynapsis and contributes to the correct localization of ATR at unsynapsed chromosomal regions (arrow 5). At the unsynapsed chromosomes, ATR phosphorylates H2AX to promote MSUC (arrow 6), as well as HORMAD1/2 (arrow 7) and SMC3 (arrow 8). Phosphorylated HORMAD1/2 further stabilizes ATR (arrow 9) at unsynapsed chromosomes and ATR further phosphorylates HORMAD1/2 (arrow 10), amplifying the unsynapsis signal via the positive feedback loop (arrow 9 and 10). (B) The status of chromosome synapsis can be indicated by the presence or absence of HORMAD1/2 and phosphorylation of HORMAD1 and SMC3. At unsynapsed chromosomal regions, the chromosome axis contains the S/T-Q motif-phosphorylated forms of HORMAD1/2 and SMC3. When homologs are synapsed, HORMAD1/2 and the Ser^1083^-phosphorylated form of SMC3 are displaced from the chromosome axis. After desynapsis, HORMAD1/2 is again included in the chromosome axis but HORMAD1 (and possibly HORMAD2) is not phosphorylated at the S/T-Q motif. Distribution of the phosphorylated forms of other components of the chromosome axis remains to be determined.

The cohesin complex is one of the important factors in DNA damage response pathways [52]. SMC1α and SMC3 are phosphorylated at S/T-Q motifs by ATM/ATR and these phosphorylation events are crucial for the DNA damage checkpoint at the intra-S phase of mitosis [28]. As in mitotic cells, SMC3 may be phosphorylated primarily in response to DSBs that are introduced by SPO11 (Figure 8A, arrow 4). Since DSBs are processed and repaired by recombination on the chromosome axis, SMC3 phosphorylation may reflect the progression of this process and be involved in DNA damage repair or checkpoints as in mitotic cells. The Ser^1083^-phosphorylated form of SMC3 is also detected at the diplotene stage on the XY chromosomes where DSBs are repaired. This phosphorylation suggests that SMC3 is additionally phosphorylated at unsynapsed regions by ATR in a manner similar to H2AX in the MSUC pathway (Figure 8A, arrow 8). To summarize, SMC3 may change the modification status according to the progression of recombination and synapsis.

### Phosphorylation of HORMAD1 and HORMAD2 may be part of a surveillance system monitoring synapsis

HORMAD1 has multiple phosphorylation sites, including Ser^375^ and a non-S/T-Q site, which are differently regulated. HORMAD1 is associated with unsynapsed and desynapsed chromosome axes [26], [27], but the Ser^375^-phosphorylated form of HORMAD1 is restricted to unsynapsed chromosomes. Collectively, our results show that HORMAD1 is phosphorylated at a non-S/T-Q site in the nucleoplasm, as well as on the chromosome, and that HORMAD1 is further phosphorylated at Ser^375^ on unsynapsed chromosomes in a SPO11-dependent manner.

HORMAD2 also has multiple phosphorylation sites. One phosphorylated form of HORMAD2 contains phosphorylation possibly at an S/T-Q site, which is regulated in a manner temporally and genetically similar to phosphorylation of HORMAD1 at Ser^375^. The other phosphorylated form of HORMAD2 is temporally regulated to take place at the late pachytene stage. Considering the localization of HORMAD2 at the unsynapsed chromosome axis during the leptotene to pachytene stages [27], we infer that HORMAD2 is primarily phosphorylated on unsynapsed chromosomes probably at an S/T-Q site similarly to Ser^375^ of HORMAD1 and that additional phosphorylation might occur on the XY chromosomes at the late pachytene stage.

ATR is recruited to unsynapsed chromosomal regions, to which HORMAD1 and HORMAD2 are localized, and phosphorylates histone H2AX, leading to MSUC [10]. Recent studies using *Hormad1*-deficient mice revealed that HORMAD1 has multiple functions, one of which is to load ATR onto the chromosome [16], [38]. We found here that phosphorylation of HORMAD1 at Ser^375^ and that of HORMAD2 are reduced in *Spo11* ^−/−^, *Brca1* ^Δ11/Δ11^ and *Sycp3* ^−/−^ spermatocytes. Intriguingly, the three mutants exhibit a similar defect in which ATR and γH2AX fail to localize to unsynapsed chromosomal regions and instead assemble at aberrant nuclear sites (Figure 7) [31]. This phenotypic similarity leads us to propose a model in which phosphorylation of HORMAD1 and HORMAD2 is required for the distribution of ATR at unsynapsed chromosomal regions (Figure 8A). HORMAD1 is primarily required for the loading of ATR irrespective of its phosphorylation state, because pseudo-sex body is formed in the *Spo11* mutant in a HORMAD1-dependent manner [16]. Therefore, HORMAD1/2 phosphorylation is dispensable for the loading of ATR, but may regulate its distribution on the prophase I chromosome. It is possible that ATR tends to aggregate at certain domains on chromosomes, as seen in the pseudo-sex body formation. Phosphorylation of HORMAD1/2 may increase the affinity of HORMAD1/2 for ATR or ATR activators, leading to the anchoring of the ATR activity at entire unsynapsed chromosomes, against this tendency. This model explains why γH2AX is localized to the unsynapsed XY chromosomes but not to the desynapsed autosomes at the diplotene stage [39], despite the presence of HORMAD1/2 at both unsynapsed and desynapsed chromosomes. Phosphorylation-based regulation of checkpoint proteins is also known for other HORMA domain-containing proteins, such as yeast Hop1 in the pachytene checkpoint [49] and mammalian MAD2 in the spindle checkpoint [53]. Thus, phosphorylation of HORMAD1/2 may regulate phosphorylation-dependent protein-protein interactions to recruit or anchor proteins involved in synapsis surveillance processes to unsynapsed chromosomes. HORMAD1/2 phosphorylation may also recruit proteins that promote SC formation, since synapsis is defective in *Hormad1*-deficient mice [16], [38]. In addition, phosphorylation of HORMAD1/2 possibly regulates inter-homolog partner choice in meiotic recombination like yeast Hop1, because this regulation appears to be impaired in the *Sycp3* mutant [54].

### Axis marks: The meiotic chromosome is spatially and temporally regulated through post-translational modifications and compositional changes of chromosome axis components

The presence or absence of HORMAD1 and HORMAD2 can distinguish whether homologs are not synapsed (unsynapsed and desynapsed) or synapsed, respectively [26], [27]. We show here that the presence or absence of the Ser^375^-phosphorylated form of HORMAD1 and the Ser^1083^-phosphorylated form of SMC3 can distinguish whether homologs that are not synapsed are unsynapsed or desynapsed, respectively. These findings prompt us to propose that modification status and composition of proteins that constitute the chromosome axis can label chromosomal regions according to the meiotic stage or progression of chromosomal events (Figure 8B). In support of this, a cohesin subunit, RAD21L, is replaced by another subunit, RAD21, in response to completion of recombination at the late pachytene stage [19], [20]. Our proposal is analogous to current models of how histone modifications and variations, which label certain chromatin regions according to DNA damage status and transcriptional activity, contribute to the recruitment of proteins involved in DNA repair, DNA-damage checkpoints and transcriptional regulation. Similarly, combinations of modifications and compositions of chromosome axis components may serve as landmarks for recruitment of proteins involved in recombination, SC formation and checkpoint control.

Our findings shed light on regulations of meiotic chromosomal events through phosphorylation of chromosome axis components. In addition to phosphorylation, other modifications of axis components may mark certain chromosomal regions to regulate meiotic events. Indeed, in yeast, SUMOylation of AE protein(s) regulates recombination and synapsis [55]. Further identification of modification sites and modification enzymes will provide more insights into regulation through the axis marks.

## Materials and Methods

### Animals

Wild-type C57BL/6 and mutant mice were used in accordance with regulations provided by the animal ethics committee of Karolinska Institutet. The *Trip13* [56], *Atm* [29], *Brca1* [57], *Spo11* [2], *Sycp3* [24], *Smc1β* [35], *Sycp1* [33] and *Tex12* [34] mutants were reported previously.

### Antibodies

To generate a phospho-specific antibody for Ser^375^ of HORMAD1 (pS375), rabbits were immunized with a Ser^375^-phosphorylated peptide corresponding to amino acids 372–382 of mouse HORMAD1. The anti-pS375 antisera were passed through a column conjugated with the non-phosphorylated peptide to remove fractions cross-reacting with non-phosphorylated HORMAD1. The flow-through fractions were then subjected to affinity-purification using the phosphorylated peptide. The purified antibody was further passed through a column conjugated with the non-phosphorylated peptide. The flow-through fractions were collected and concentrated by ultrafiltration (Amicon, Millipore). The following antibodies were also used: guinea pig anti-SYCP2, anti-SMC1β, anti-STAG3, anti-REC8 and anti-SYCP1 antibodies [58]; guinea pig anti-HORMAD1antibody [26]; rabbit anti-HORMAD1 antibody (13917-1-AP) from Proteintech Group; rabbit anti-pS/T-Q antibody (#2851) from Cell Signaling Technology; rabbit anti-pS1083 antibodies (A300-480A and IHC-00070) from Bethyl Laboratories; mouse and rabbit anti-γH2AX antibodies (#05-636 and #07-164) from Millipore; mouse anti-SYCP3 (sc-74569), rabbit anti-HORMAD2 (sc-82192), goat anti-SMC3 (sc-8198) and goat anti-ATR (sc-1887) antibodies from Santa Cruz Biotechnology; rabbit anti-SMC3 (ab9263) and rabbit anti-SYCP1 (ab15090) antibodies from Abcam; mouse anti-SYCP1 antibody (a gift from C. Heyting).

### Immunoblotting and immunoprecipitation

Testis nuclear extracts were prepared as described previously [26]. For phosphatase treatment, nuclear extracts were incubated with λ-phosphatase (New England Biolabs) in the presence or absence of phosphatase inhibitor cocktail (Merck) for 90 min at 30°C. For fractionation of nuclear extracts, testes were homogenized in a buffer containing 0.32 M Sucrose, 10 mM HEPES pH 7.4, 1 mM phenylmethylsulfonyl fluoride (PMSF) and the complete protease inhibitor cocktail (Roche). After centrifugation at 1000 *g*, the pellet was suspended in a buffer containing 25 mM Tris-HCl pH 7.5, 150 mM NaCl, 5 mM EDTA, 1% Triton X-100, 0.5% Na-deoxycholate, 0.1% SDS and protease inhibitors. After centrifugation at 16000 *g*, the supernatant was collected as a soluble fraction. The pellet was resuspended in the same buffer. After sonication and centrifugation at 16000 *g*, the supernatant was recovered as an insoluble fraction. Proteins were separated on a 5, 8, or 10% polyacrylamide gel to detect slow-migrating forms or on a 4–12% NuPAGE Bis-Tris gel (Invitrogen), and were subsequently transferred onto an Immobilon-P membrane (Millipore). Immunoprecipitation was performed as described previously [26].

### Immunofluorescence staining

For preparation of nuclear spreads, a drying-down technique [59] was used. Indirect immunofluorescence analysis was performed using previously described antibodies [26]. We also used the following primary antibodies and dilutions: anti-pS375, 1∶100; anti-pS1083, 1∶100; anti-γH2AX, 1∶400; anti-ATR, 1∶50. Slides were viewed at room temperature using Leica DMRA2 and DMRXA microscopes. Images were captured with a Hamamatsu digital charge-coupled device camera C4742-95 and processed with Openlab 3.1.4 software (Improvision) and Adobe Photoshop.

## Supporting Information

Figure S1 Meiotic chromosome axis proteins were analyzed as in Figure 1A. The positions of molecular weight markers are presented on the left.
(TIF)

Figure S2 HORMAD1 and SMC3 are phosphorylated at unsynapsed chromosome axes. (A and B) Nuclear spreads of oocytes from embryonic ovaries were analyzed by immunostaining. The Ser^375^-phosphorylated form of HORMAD1 (A) and the Ser^1083^-phosphorylated form of SMC3 (B) were labeled by phosphorylation-specific antibodies. Chromosome axes were labeled by SYCP3 and unsynapsed and desynapsed axes were marked by HORMAD1. Arrows indicate synapsed regions of homologs. Arrowheads indicate desynapsed regions of homologs. Bars, 10 µm.
(TIF)

Figure S3 SMC3 is phosphorylated during prophase I. (A) Testis nuclear extracts from juvenile mice of each age were immunoprecipitated with the anti-SMC3 antibody. 80% of the immunoprecipitated SMC3 and the rest were separated on a gradient gel and immunoblotted with antibodies against the Ser^1083^-phosphorylated form of SMC3 (pS1083) and normal SMC3, respectively. (B) Nuclear spreads of spermatocytes were labeled with anti-pS1083, anti-SMC3 and anti-SYCP3 antibodies. Arrowheads indicate the XY bivalent. Bars, 10 µm.
(TIF)

Figure S4 HORMAD1 and SMC3 are phosphorylated on unsynapsed chromosomes in the absence of ATM. (A) Nuclear spreads of *Atm* ^−/−^ zygotene-like spermatocytes were labeled with anti-pS375, anti-SYCP3 and anti-SYCP1 antibodies. (B) Nuclear spreads of *Atm* ^−/−^ zygotene-like spermatocytes were labeled with anti-pS1083, anti-SYCP3 and anti-SYCP1 antibodies. Bars, 10 µm.
(TIF)

Figure S5 Localization of the Ser^375^-phosphorylated form of HORMAD1 and ATR during the prophase I stage of meiosis. Nuclear spreads of spermatocytes were labeled with anti-pS375, anti-SYCP3 and anti-ATR antibodies. Arrowheads indicate the XY bivalent. Bars, 10 µm.
(TIF)

Figure S6 HORMAD1 is phosphorylated on unsynapsed chromosomes in the absence of SYCP1, TEX12 or SMC1β. (A–C) Nuclear spreads of *Sycp1* ^−/−^ (A), *Tex12* ^−/−^ (B) and *Smc1β* ^−/−^ (C) zygotene-like spermatocytes were labeled with anti-pS375, anti-SYCP3 and anti-HORMAD1 antibodies. Bars, 10 µm.
(TIF)

Figure S7 Phosphorylation of HORMAD1 and SMC3 in the absence of SYCP3. (A) Nuclear spreads of *Sycp3* ^−/−^ zygotene-like spermatocytes were labeled with anti-pS375 and anti-REC8 antibodies. (B) Nuclear spreads of *Sycp3* ^−/−^ zygotene-like spermatocytes were labeled with anti-HORMAD1 and anti-REC8 antibodies. HORMAD1 is loaded on cohesin cores. (C) Nuclear spreads of *Sycp3* ^−/−^ zygotene-like spermatocytes were labeled with anti-pS1083 and anti-REC8 antibodies. The Ser^1083^-phosphorylated form of SMC3 was detected as foci along the discontinuous cohesin cores. Bars, 10 µm.
(TIF)
