# Supplementary figures and images for: Phosphorylation of Chromosome Core Components May Serve as Axis Marks for the Status of Chromosomal Events during Mammalian Meiosis
Source: PLoS Genet. 2012 Feb 9;8(2):e1002485. doi: 10.1371/journal.pgen.1002485 (PMC3276554; doi:10.1371/journal.pgen.1002485)

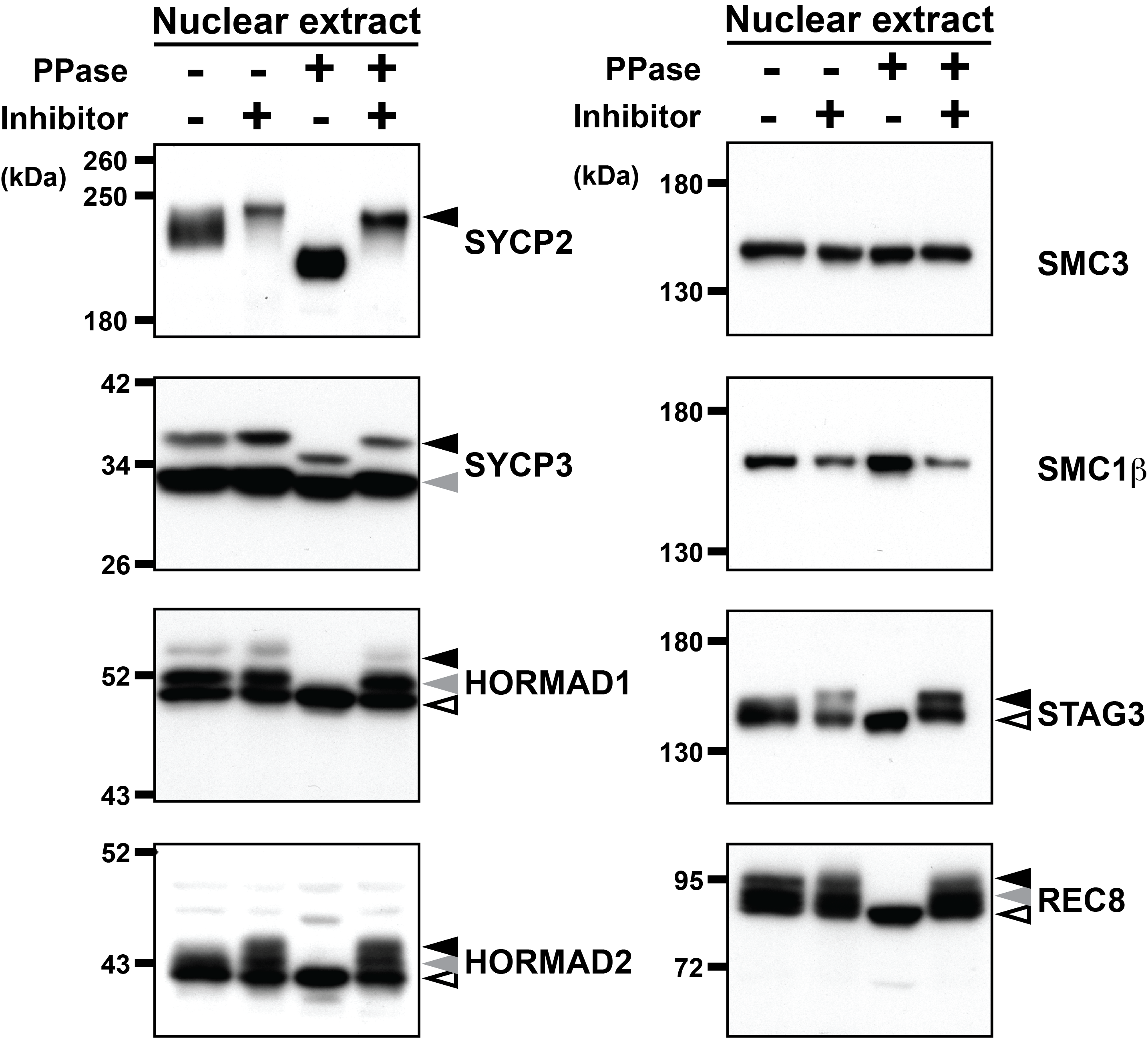

Supplement: Figure S1 — Meiotic chromosome axis proteins were analyzed as in Figure 1A. The positions of molecular weight markers are presented on the left. (TIF) [file pgen.1002485.s001.tif]

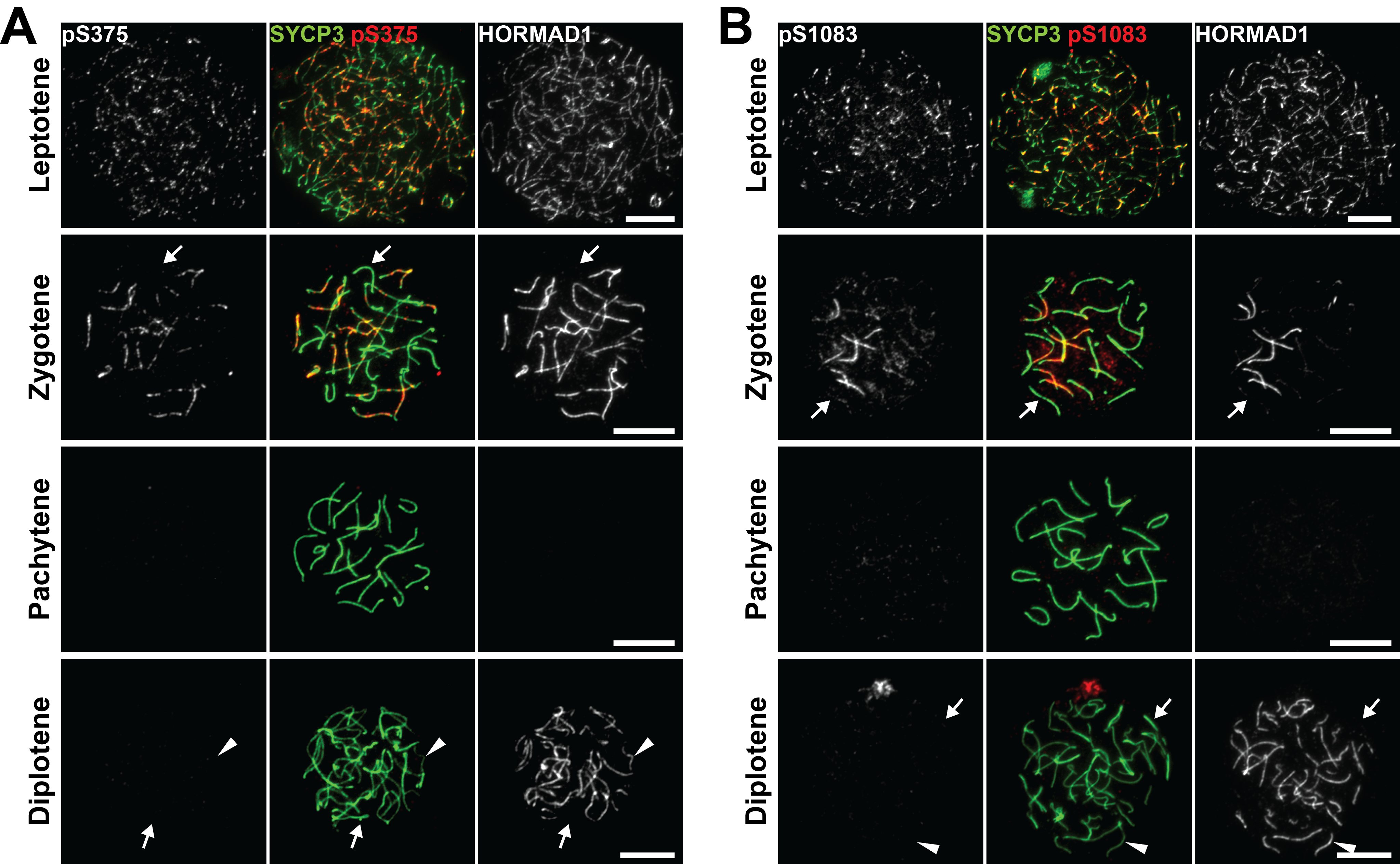

Supplement: Figure S2 — HORMAD1 and SMC3 are phosphorylated at unsynapsed chromosome axes. (A and B) Nuclear spreads of oocytes from embryonic ovaries were analyzed by immunostaining. The Ser375-phosphorylated form of HORMAD1 (A) and the Ser1083-phosphorylated form of SMC3 (B) were labeled by phosphorylation-specific antibodies. Chromosome axes were labeled by SYCP3 and unsynapsed and desynapsed axes were marked by HORMAD1. Arrows indicate synapsed regions of homologs. Arrowheads indicate desynapsed regions of homologs. Bars, 10 µm. (TIF) [file pgen.1002485.s002.tif]

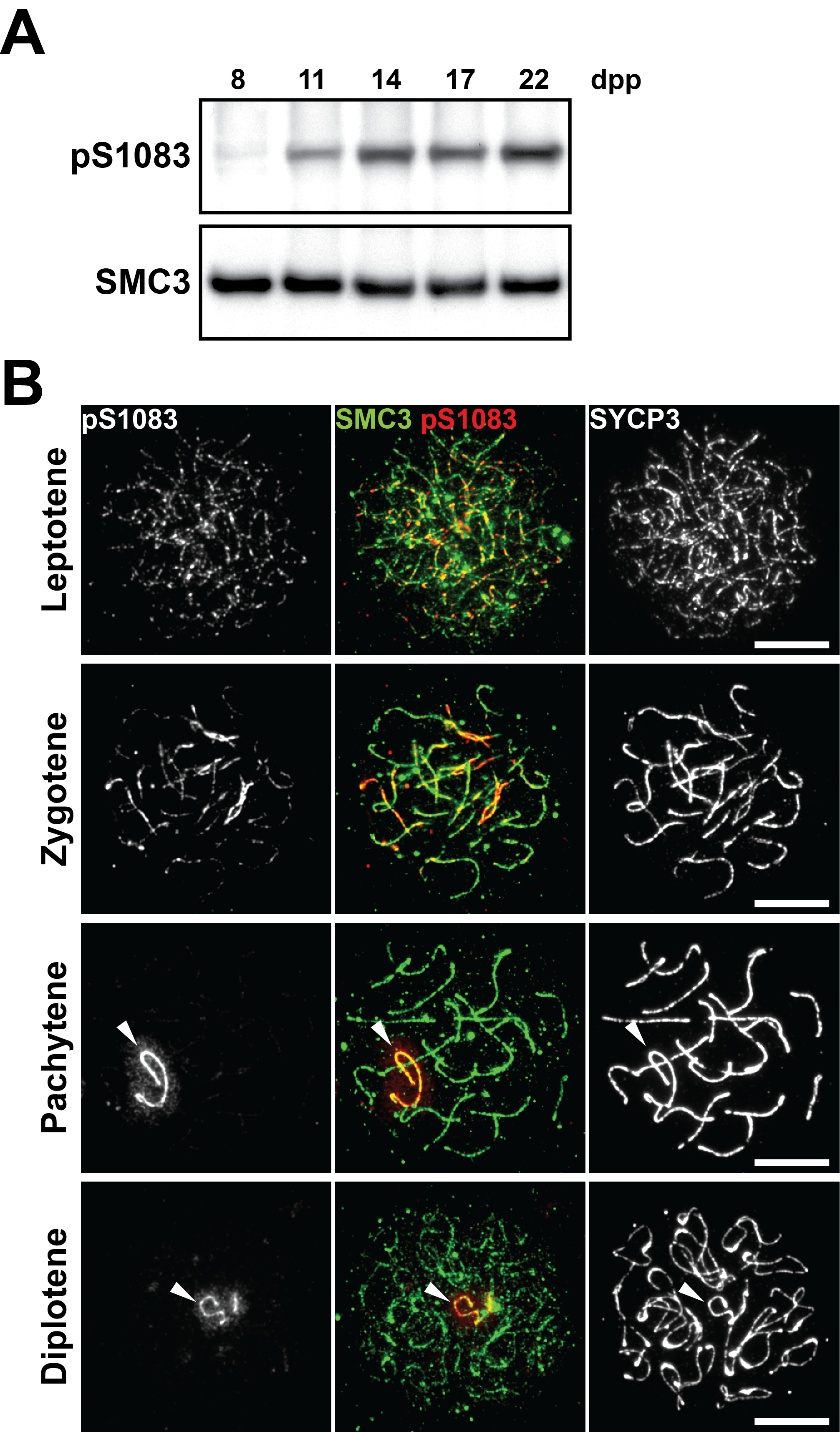

Supplement: Figure S3 — SMC3 is phosphorylated during prophase I. (A) Testis nuclear extracts from juvenile mice of each age were immunoprecipitated with the anti-SMC3 antibody. 80% of the immunoprecipitated SMC3 and the rest were separated on a gradient gel and immunoblotted with antibodies against the Ser1083-phosphorylated form of SMC3 (pS1083) and normal SMC3, respectively. (B) Nuclear spreads of spermatocytes were labeled with anti-pS1083, anti-SMC3 and anti-SYCP3 antibodies. Arrowheads indicate the XY bivalent. Bars, 10 µm. (TIF) [file pgen.1002485.s003.tif]

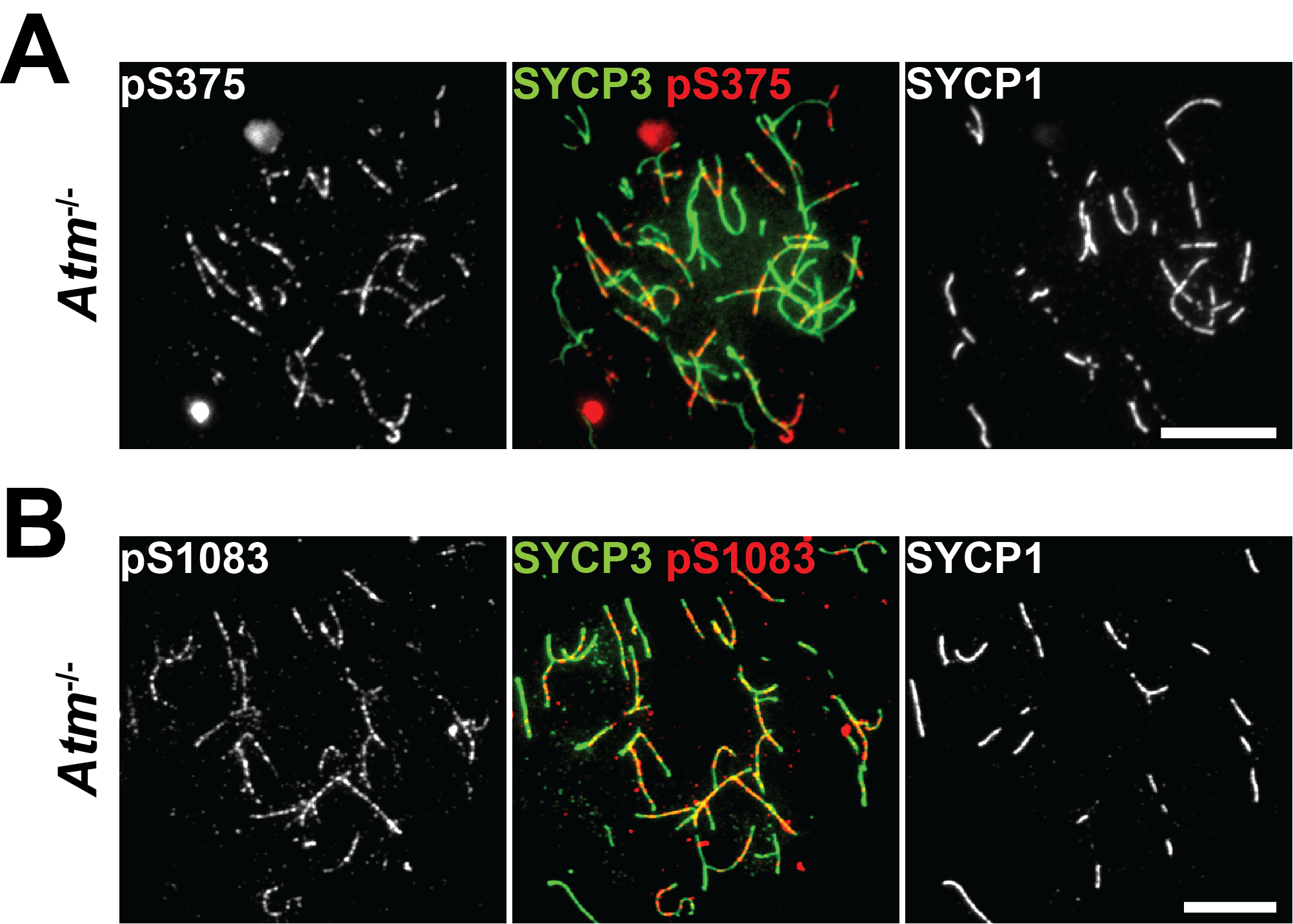

Supplement: Figure S4 — HORMAD1 and SMC3 are phosphorylated on unsynapsed chromosomes in the absence of ATM. (A) Nuclear spreads of Atm −/− zygotene-like spermatocytes were labeled with anti-pS375, anti-SYCP3 and anti-SYCP1 antibodies. (B) Nuclear spreads of Atm −/− zygotene-like spermatocytes were labeled with anti-pS1083, anti-SYCP3 and anti-SYCP1 antibodies. Bars, 10 µm. (TIF) [file pgen.1002485.s004.tif]

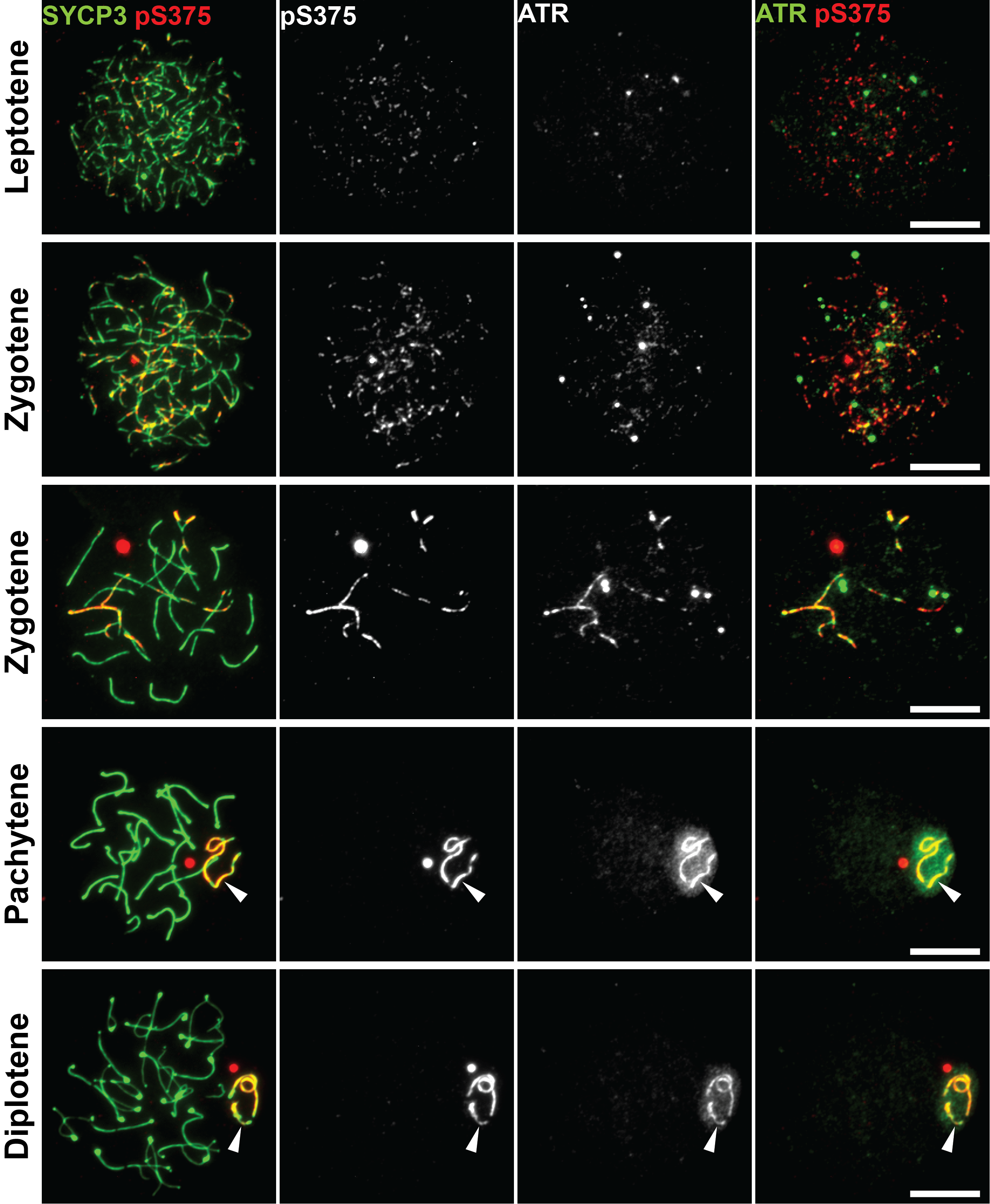

Supplement: Figure S5 — Localization of the Ser375-phosphorylated form of HORMAD1 and ATR during the prophase I stage of meiosis. Nuclear spreads of spermatocytes were labeled with anti-pS375, anti-SYCP3 and anti-ATR antibodies. Arrowheads indicate the XY bivalent. Bars, 10 µm. (TIF) [file pgen.1002485.s005.tif]

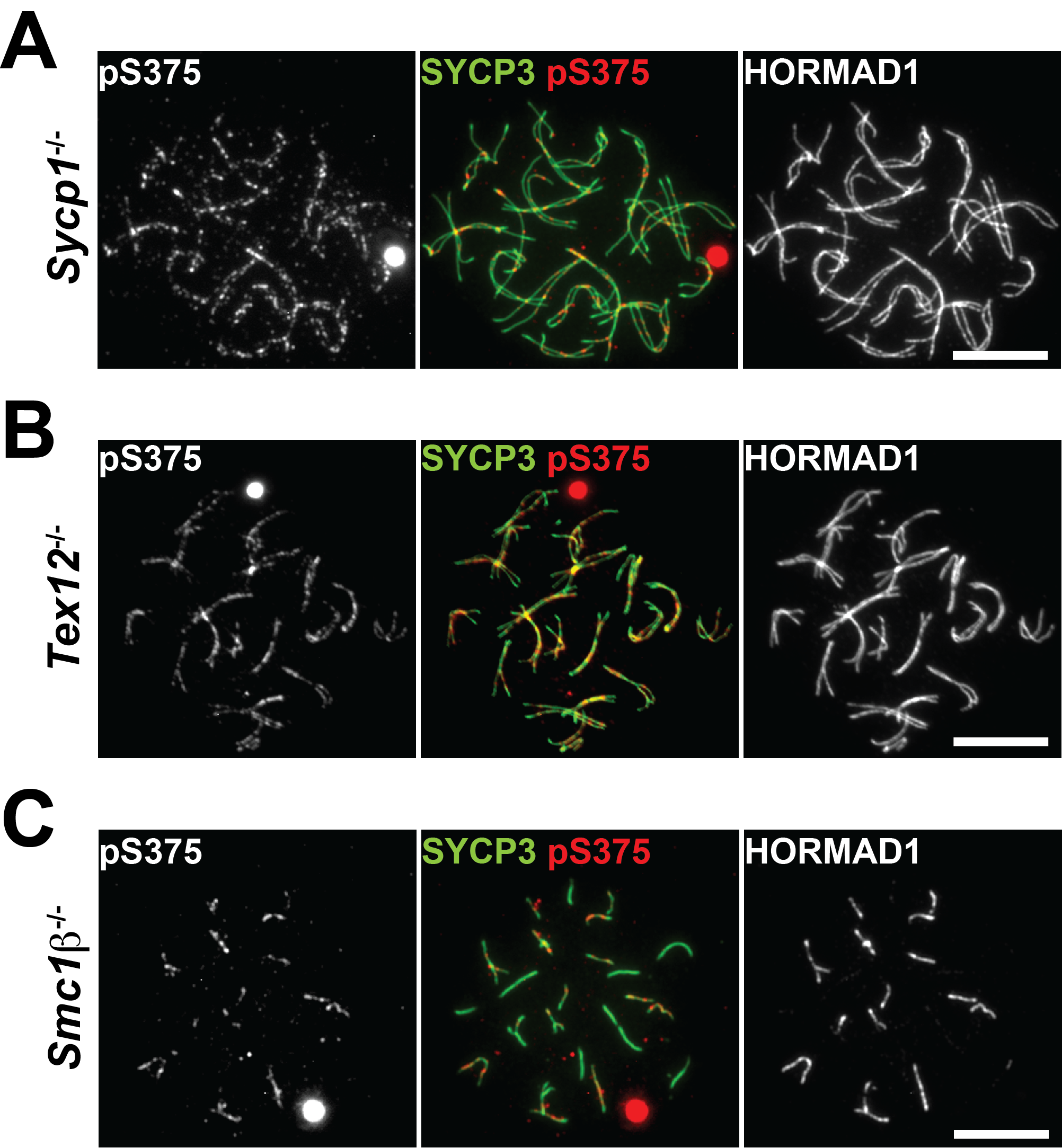

Supplement: Figure S6 — HORMAD1 is phosphorylated on unsynapsed chromosomes in the absence of SYCP1, TEX12 or SMC1β. (A–C) Nuclear spreads of Sycp1 −/− (A), Tex12 −/− (B) and Smc1β −/− (C) zygotene-like spermatocytes were labeled with anti-pS375, anti-SYCP3 and anti-HORMAD1 antibodies. Bars, 10 µm. (TIF) [file pgen.1002485.s006.tif]

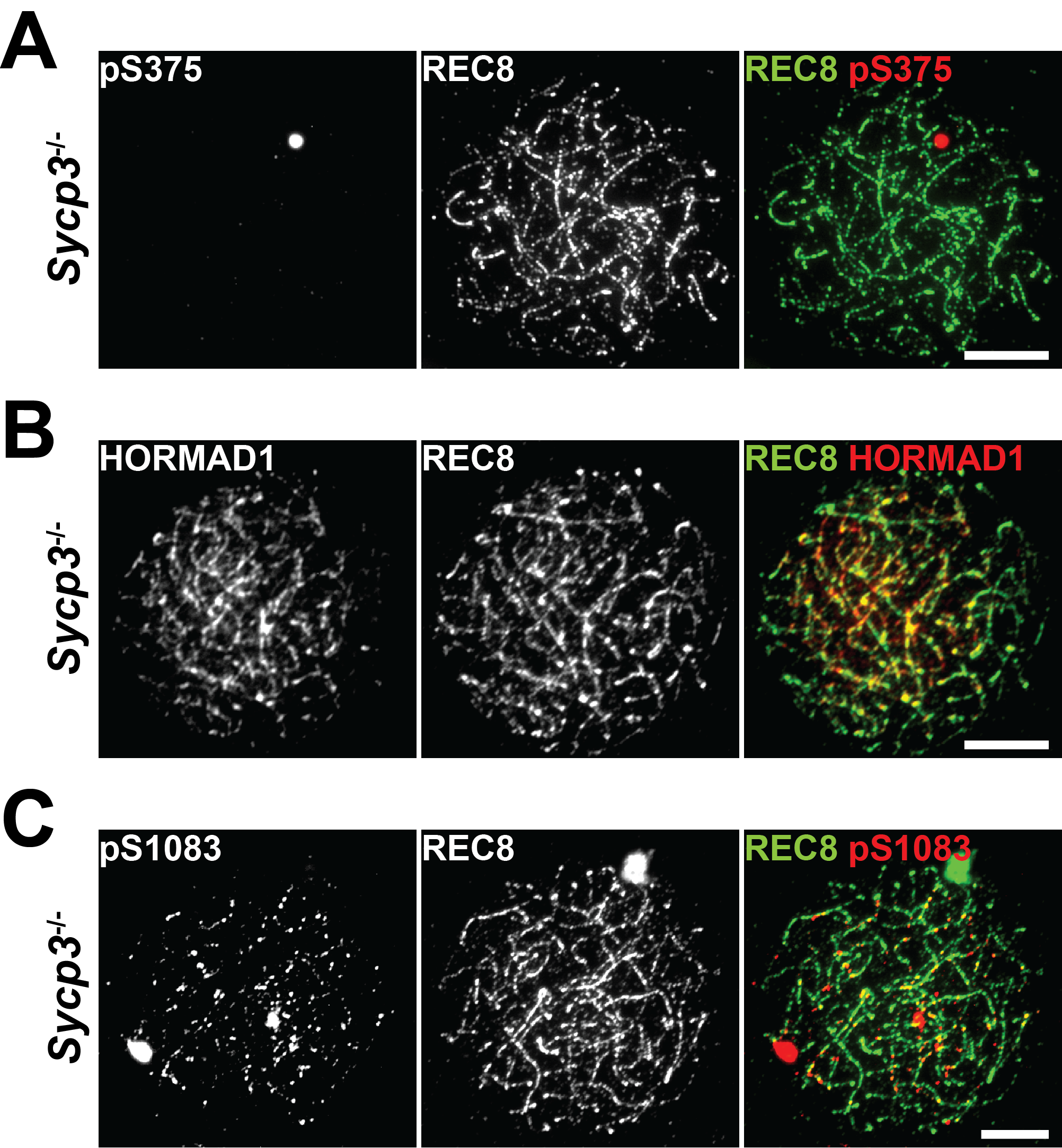

Supplement: Figure S7 — Phosphorylation of HORMAD1 and SMC3 in the absence of SYCP3. (A) Nuclear spreads of Sycp3 −/− zygotene-like spermatocytes were labeled with anti-pS375 and anti-REC8 antibodies. (B) Nuclear spreads of Sycp3 −/− zygotene-like spermatocytes were labeled with anti-HORMAD1 and anti-REC8 antibodies. HORMAD1 is loaded on cohesin cores. (C) Nuclear spreads of Sycp3 −/− zygotene-like spermatocytes were labeled with anti-pS1083 and anti-REC8 antibodies. The Ser1083-phosphorylated form of SMC3 was detected as foci along the discontinuous cohesin cores. Bars, 10 µm. (TIF) [file pgen.1002485.s007.tif]
